# Protocol for multiscale *in vivo* adenosine triphosphate imaging in living mice

**DOI:** 10.1016/j.xpro.2026.104958

**Published:** 2026-11-10

**Authors:** Ryosuke Ochi, Masamichi Yamamoto

**Affiliations:** 1 Department of Research Promotion and Management, National Cerebral and Cardiovascular Center, Kishibe-Shimmachi, Suita, Osaka 564-8565, Japan

**Keywords:** Metabolism, Model Organisms, Protocols in Metabolomics and Lipidomics

## Abstract

*In vivo* adenosine triphosphate (ATP) imaging is a powerful approach for studying physiological and pathological mechanisms underlying various metabolic processes. Here, we present a protocol for multiscale *in vivo* ATP imaging using AVID (ATP visualization *in vivo* directly) mice, which express GO-ATeam2, a fluorescent biosensor. We describe steps for endotracheal intubation, mechanical ventilation, and imaging preparation. We then detail procedures for macroscopic or two-photon imaging and image analysis.

## Highlights

• Intravital macroscopic ATP imaging across multiple thoracic and abdominal organs
• Cellular-resolution ATP monitoring in mouse liver using two-photon microscopy
• Tissue stabilization via cyanoacrylate-glued washer minimizes motion artifacts
• Step-by-step workflow for ratiometric FRET calculation and ratio image rendering

## Abstract (editor highlights)

Publisher’s note: Undertaking any experimental protocol requires adherence to local institutional guidelines for laboratory safety and ethics.

## Graphical abstract

**Figure:**
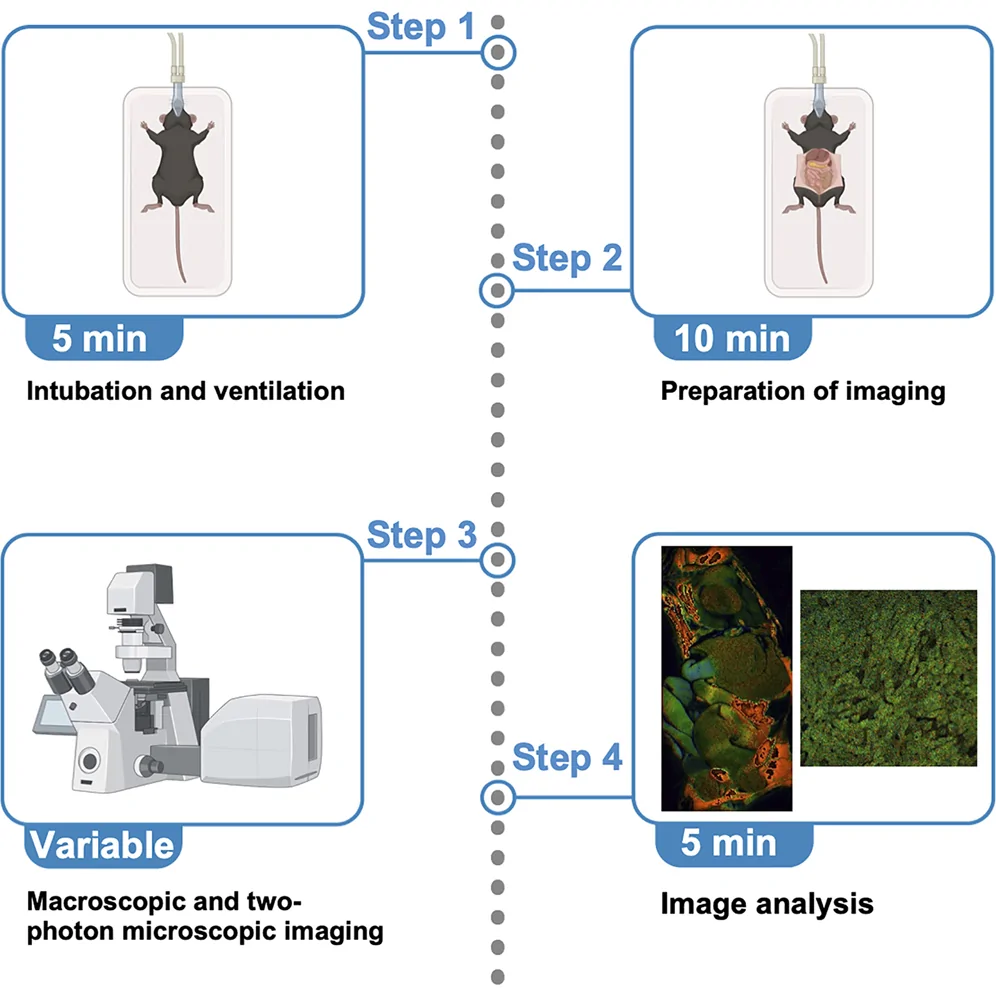

## Before you begin

This protocol describes the procedures for *in vivo* imaging of intra-abdominal and intrathoracic organs to measure intracellular adenosine triphosphate (ATP) levels in healthy mice. We have also applied this protocol to a model of acute myocardial infarction.^1^ Furthermore, this protocol is applicable to a variety of mouse models, making it a valuable tool for investigating energy metabolism under both physiological and diverse pathological conditions.

### Breeding mice

**Timing: Variable**

1. Prepare mutant AVID mice.
  a. Breed AVID mice with target mutants or wild-type mice.
  b. Verify the expression of GO-ATeam2 using an LED excitation light or confirm the insertion of GO-ATeam2 via PCR genotyping.
    ***Note:*** For screening purposes, biosensor expression is verified by the presence of fluorescence, not by ratiometric measurement; therefore, precise matching of the excitation wavelength to the absorption maximum is not required. Although the biosensor signal can be detected with a GFP excitation light, an RFP excitation light provides superior visibility for screening because it directly excites the Kusabira Orange acceptor and reduces tissue autofluorescence.
    ***Note:*** Both homozygous and heterozygous AVID mice can be used; however, homozygous mice are recommended because their higher fluorescence intensity provides a better signal-to-noise ratio.
  c. Raise the offspring until they reach the appropriate age for each experiment.
    ***Note:*** GO-ATeam2 is constitutively expressed under a ubiquitous promoter across all tissues and developmental stages, and AVID mice of either sex can be used. The ventilation procedures described in this protocol were established for post-weaning and adult mice; in our experiments, mice with a body weight of approximately 15–40 g were used.

### Innovation

Conventionally, ATP levels have been measured *in vivo* using nuclear magnetic resonance spectroscopy and bioluminescence imaging. However, these methods lack sufficient spatial and temporal resolution to assess ATP fluctuations. Tissue energy metabolism, including that of the liver, can also be assessed without any reporters or labels—for example, by autofluorescence imaging of endogenous coenzymes (NAD(P)H and FAD), fluorescence lifetime imaging microscopy,^2^ and mass spectrometry imaging.^3^ Nevertheless, these label-free modalities report surrogate metabolic indices, such as the cellular redox state, rather than ATP itself, or require tissue destruction, and therefore cannot capture real-time ATP dynamics at cellular resolution in intact organs. To address these limitations, several genetically encoded biosensors have been developed to measure ATP levels in living cells.^4^

GO-ATeam is an ATP biosensor based on Förster resonance energy transfer (FRET) that exhibits high sensitivity and robustness against acidification.^5^ We developed a transgenic mouse model expressing GO-ATeam2 under a ubiquitous promoter, designated as AVID (ATP Visualization *In vivo* Directly) mice. This protocol describes imaging methods using AVID mice to monitor intracellular ATP levels *in vivo*.

### Institutional permissions

All experiments were approved by the Institutional Animal Care and Use Committee of the National Cerebral and Cardiovascular Center, Gunma University, and Kyoto University. All procedures described in this protocol are terminal. Readers must acquire appropriate permissions from their respective institutions prior to executing this protocol.

## Key resources table

**Table:** 

REAGENT or RESOURCE	SOURCE	IDENTIFIER
**Chemicals, peptides, and recombinant proteins**		

Meloxicam	Boehringer Ingelheim	Metacam
Isoflurane	VIATRIS	N/A

**Experimental models: Organisms/strains**		

Mouse: AVID, B6.Cg-*Gt(ROSA)26Sor*^*tm1.1(GO-ATeam2)*^ (homozygous or heterozygous; male and female; post-weaning to adult)	Ohnishi et al.^1^/The Jackson Laboratory	JAX stock no.041022; MTA required

**Software and algorithms**		

ImageJ	NIH	https://imagej.nih.gov/ij/
MetaMorph	Molecular Devices	N/A
HCImage, ver. 4.2	Hamamatsu Photonics	N/A
Las X, ver. 3.5.5	Leica Microsystems	N/A
Microsoft Excel, ver. 16	Microsoft	N/A
StackReg	Thevenaz et al.^6^	https://bigwww.epfl.ch/thevenaz/stackreg/

**Other**		

Fluorescence macroscope (M165FC)	Leica Microsystems	N/A
Objective lens 0.63× PLAN-APO	Leica Microsystems	10450027
Objective lens 1.0× PLAN-APO	Leica Microsystems	10450028
Modified GFP filter (excitation filter only; emission filter removed)	Leica Microsystems	N/A
Optical Splitter	Hamamatsu Photonics	W-VIEW GEMINI
DCLP dichroic mirror (540DCLP)	Chroma Technology	540DCLP-1826
Emission filter (515/30)	Chroma Technology	515/30
Emission filter (575/40)	Chroma Technology	575/40
Digital CMOS camera	Hamamatsu Photonics	ORCA-flash4.0 v2
External trigger cable	Hamamatsu Photonics	A12107-05
LED light source	Excelitas Technologies	X-Cite 110LED
Inverted two-photon microscope (TCS SP8 MP)	Leica Microsystems	N/A
Integrated epifluorescence illumination with RFP filter set	Leica Microsystems	N/A
Mai Tai HP	Spectra-Physics	N/A
Water-immersion objective lens HC FLUOTAR L25x/0.95 W VISIR	Leica Microsystems	11506375
Anesthesia unit	Natsume Seisakusho	KN-1071
Custom-made nose cone (modified 5 mL syringe)	TERUMO	N/A
Warm plate	Kitazato Corporation	KM-1
Stage heater	Tokai Hit	TP-SQH-B15Q
Ventilator	Natsume Seisakusho	KN-58
Vertical support	Custom-made	N/A
Intravenous cannula (20 G, 32 mm)	TERUMO	SR-OT2032C
PIPETMAN Classic P20 (2–20 μL)	Gilson	F123600
Eclipse 200 μL pipet tips	Labcon	1093-260
Tissue-grade cyanoacrylate instant adhesive	Daiichi Sankyo	Aron Alpha A “Sankyo”
Surgical scissors	Leprex	LB-12
Electric cautery	Geiger	TCU-150
Cover slip	Matsunami Glass	C025601
Polycarbonate washer	Hirosugi Keiki	PCW-0310-03

## Step-by-step method details

### Endotracheal intubation and mechanical ventilation

**Timing: 5 min**

This section describes the steps to intubate and ventilate mice for *in vivo* imaging of intrathoracic organs.

1. Prepare a ventilator.
  a. Connect the anesthesia unit to the ventilator so that room air (21% O_2_) containing 1.5%–2.0% isoflurane is supplied to the ventilator at a flow rate of 0.5 L/min; this gas mixture is used for ventilation.
  b. Set the respiratory rate to 140 breaths per minute.
  c. Set the percentage of inspiratory time to 50%.

***Note:*** The tidal volume cannot be set directly on this ventilator. Instead, the airway pressure is measured in real time by the ventilator and is approximately 1,000 Pa (∼10 cmH_2_O) under the described settings.

2. Place a mouse into an induction chamber pre-filled with 4.0% isoflurane gas.

***Optional:*** Administer an analgesic agent, such as meloxicam, subcutaneously, in accordance with institutional animal care guidelines.

3. After confirming that the mouse is deeply anesthetized, place the mouse on a vertical support in the upright position and suspend its upper incisors (Figure 1A).

**Figure 1:**
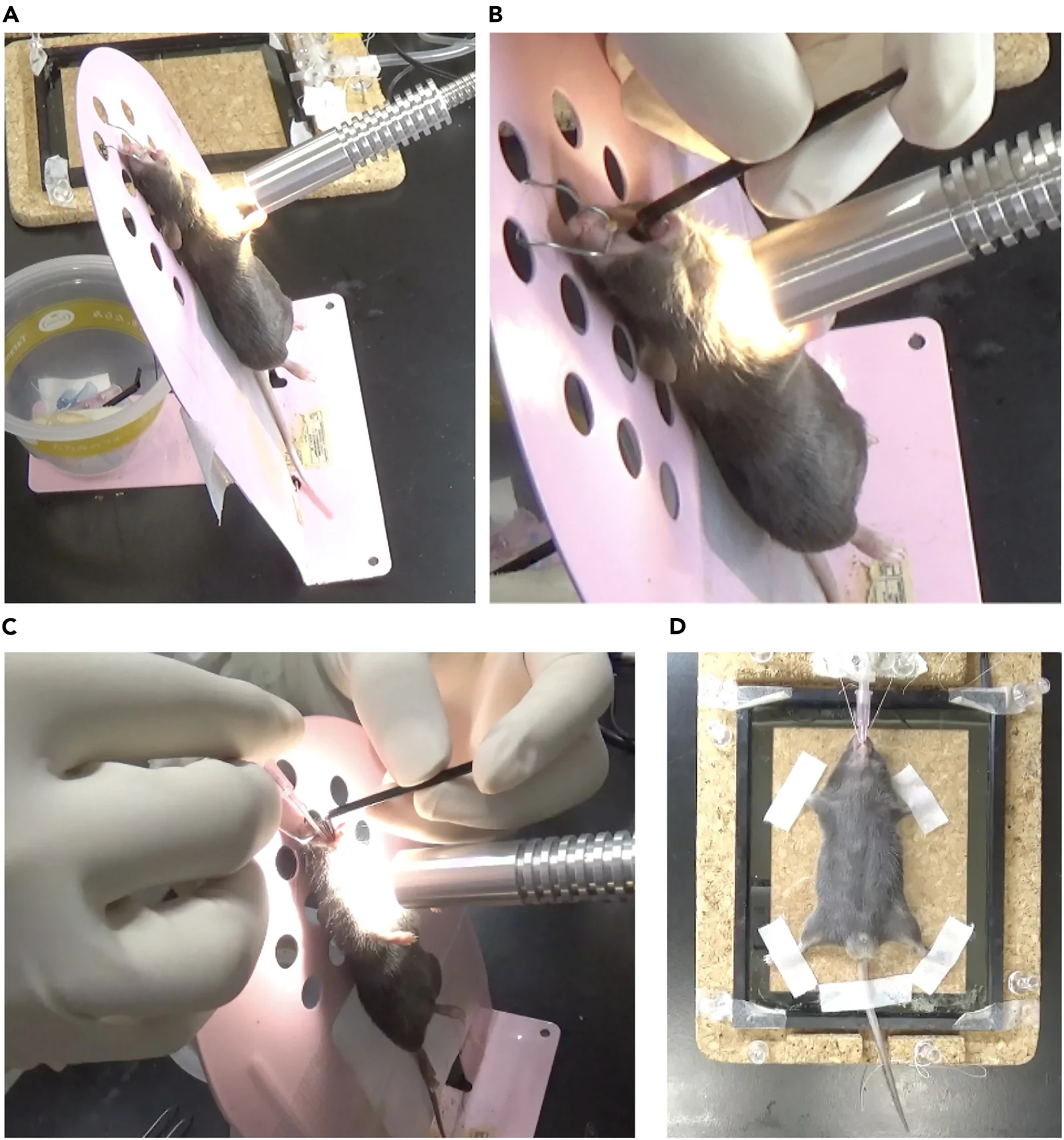
Endotracheal intubation and mechanical ventilation (A) Positioning of the mouse for intubation. (B) The tongue is retracted with a hook and the neck is transilluminated to visualize the vocal cords. (C) Insertion of an intravenous cannula into the trachea. (D) The mouse is placed on a heating pad in the supine position and the cannula is connected to the ventilator. The upper incisors are suspended with a hook, and the tail and four paws are secured with tape.
4. Illuminate the throat externally to clearly visualize the glottis.
5. Hold the tongue gently using a hook to bring the vocal cords into view (Figure 1B).
6. Insert a 20-G, 32-mm intravenous cannula (Terumo, SR-OT2032C) smoothly into the trachea (Figure 1C).
7. Place the mouse onto a heating pad in the supine position and immediately connect the cannula to the ventilator (Figure 1D).
8. Verify that the thorax expands and contracts in synchronization with the ventilator cycles.

### Exposure of intra-abdominal and intrathoracic organs for macroscopic imaging

**Timing: 10 min**

This section describes the preparations for *in vivo* imaging of intra-abdominal and intrathoracic organs.

9. Maintain anesthesia with 1.5%–2.0% isoflurane and ventilation.

***Note:*** If a sufficient depth of anesthesia is not obtained during the surgical procedures, temporarily increase the isoflurane concentration to 2.0%–2.5%, and return it to 1.5%–2.0% before starting image acquisition.

10. Place the mouse on a heat pad (37°C).
11. Suspend its upper incisors with a hook and fix its tail and four paws with a piece of tape (Figure 1D).
12. Using surgical scissors, incise the skin to expose the thorax and abdominal wall (Figure 2A).
  a. Make a coronal skin incision near the inferior margin of the costal arch.
  b. Make a longitudinal skin incision running along the midline from the costal arch to the caudal end of the abdomen.
  c. Extend the longitudinal incision up to the neck, running slightly left of the midline over the thorax.

**Figure 2:**
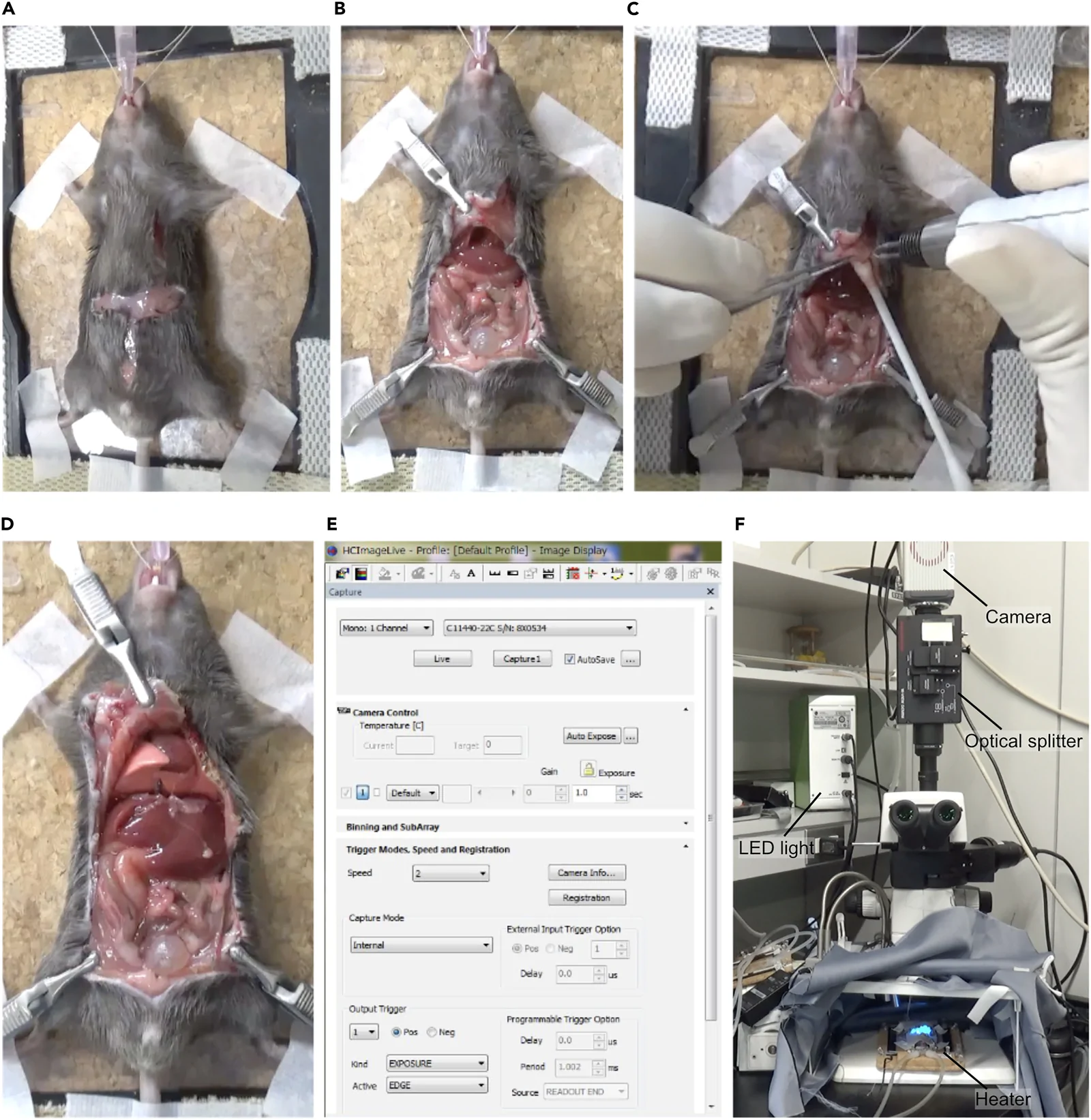
Exposure of intrathoracic and abdominal organs and the macroscopic imaging setup (A) The skin was incised coronally along the inferior margin of the costal arch and longitudinally along the midline over the abdomen, continuing slightly left of the midline over the thorax. (B) The abdominal wall was opened to expose the abdominal organs. (C) The rib cage is incised with an electric cautery. A cotton swab is inserted beneath the rib cage to protect the intrathoracic organs from the cautery. (D) The right side of the incised rib cage is retracted laterally with a clip, exposing both the intrathoracic and abdominal organs. (E) Representative application window of HCImage for macroscopic imaging. (F) The macroscopic imaging setup. The mouse is placed on a heater on the stage of a fluorescence stereo microscope. An LED light source provides fluorescence excitation, an optical splitter separates the emission into the EGFP and FRET channels, and both channels are recorded with a digital CMOS camera.
13. Make a coronal incision at the bottom of the rib cage and a sagittal incision on the midline using an electric cautery to expose the intra-abdominal organs (Figure 2B).

***Note:*** Alternatively, the abdominal wall can be opened with scissors; minor bleeding does not interfere with imaging.

14. Cut the diaphragm.
15. Make a sagittal incision on the rib cage at the left side of the midline using an electric cautery to expose the intrathoracic organs (Figure 2C).

***Note:*** Insert a cotton swab between the rib cage and intrathoracic organs to prevent the electric cautery from touching the organs.

16. Hold the rib cage and abdominal wall laterally with clips (Figure 2D).

***Note:*** An especially rapid setup is not required. As long as mechanical ventilation and body temperature are maintained, the baseline FRET/GFP ratio remains stable during the surgical preparation. Rather than speed, gentle handling of the organs is critical, because surgical trauma itself induces artificial local declines in the FRET/GFP ratio (see expected outcomes).

### Macroscopic imaging

**Timing: Variable (depending on duration of time-lapse imaging)**

This section describes the organ-level imaging procedures using a macroscopic fluorescence setup.

17. Turn on the digital CMOS camera (ORCA-flash4.0), the host PC, and the LED light source (X-Cite 110LED).
18. Launch the HCImage software and confirm that a stable connection is established with the camera hardware.
19. Set the output intensity of the X-Cite LED light source to 100%.
20. Confirm that the GFP excitation filter (ET470/40) is placed in the excitation light path.
21. Confirm that W-VIEW GEMINI image-splitting optics are equipped with a dichroic mirror (540 nm) and two emission filters (515/30 nm for the EGFP channel and 575/40 nm for the FRET channel).

***Note:*** The EGFP and FRET channels are projected side-by-side onto the same camera sensor and acquired simultaneously in a single exposure; no filter switching is required.

***Note:*** The spatial registration of the two channels is adjusted with the W-VIEW GEMINI at the initial installation, and re-verified whenever the optical configuration is changed. Misregistration between the two channels distorts the pixel-by-pixel FRET/GFP ratio, particularly at structural boundaries.

22. Configure the software acquisition settings as follows (Figure 2E):
  a. Format: 2048 × 2048 pixels.
  b. Pixel size: 23.0 × 23.0 μm.
  c. Depth: 16-bit.
  d. Exposure: 1.0 s.
  e. Zoom: 0.73×.

***Note:*** The acquisition speed should be tailored to the kinetics of the biological process of interest. For capturing rapid, sub-second ATP dynamics (e.g., skeletal muscle contraction; Figures 1G–1I of Ohnishi et al^1^; ), the macroscopic configuration is recommended. Utilizing a sCMOS camera and image-splitting optics allows simultaneous dual-channel acquisition at high frame rates (typically ∼10 frames/s with 100 ms exposure; up to 20 frames/s was used for skeletal muscle contraction^1^) while preserving a high signal-to-noise ratio.^1^

23. Select the appropriate objective lens depending on the desired field of view: use the 0.63× objective lens to capture the whole body within a single frame, or switch to the 1.0× objective lens to image a specific targeted organ area.
24. Place the ventilated mouse onto the macroscope stage (Figure 2F).
25. Manually adjust the XYZ position of the stage to align the region of interest containing the target organs.
26. Fully enclose the microscope stage area with a blackout curtain.
27. Define the parameters for time-lapse imaging.

***Note:*** The imaging duration should be determined according to the biological process of interest. As a representative example, we performed time-lapse imaging for 60 min at an acquisition interval of one frame per minute to monitor ATP dynamics during acute myocardial infarction.^1^

28. Start the imaging acquisition sequence.

***Note:*** Connect a trigger cable from the camera to the X-Cite 110LED light source to synchronize the illumination on/off cycles with the camera exposure. This setup ensures that the excitation light strikes the tissue only during active frame capture, minimizing photobleaching and phototoxicity.

### Exposure of the liver for imaging at cellular level

**Timing: 10 min**

This section outlines the surgical preparation for single-cell level *in vivo* imaging of the liver via two-photon microscopy.

29. Place the mouse into an induction chamber filled with 4.0% isoflurane gas.
30. Transfer the anesthetized mouse to a heating pad maintained at 37°C and sustain anesthesia with 1.5%–2.0% isoflurane.

***Note:*** If a sufficient depth of anesthesia is not obtained during the surgical procedures, temporarily increase the isoflurane concentration to 2.0%–2.5%, and return it to 1.5%–2.0% before starting image acquisition.

31. Secure the upper incisors with a hook and tightly fix the tail and four paws using laboratory tape.
32. Make a skin incision using standard surgical scissors to expose the abdominal wall.
33. Perform a coronal incision at the lower boundary of the rib cage using an electric cautery to expose the liver surface (Figure 3A).

**Figure 3:**
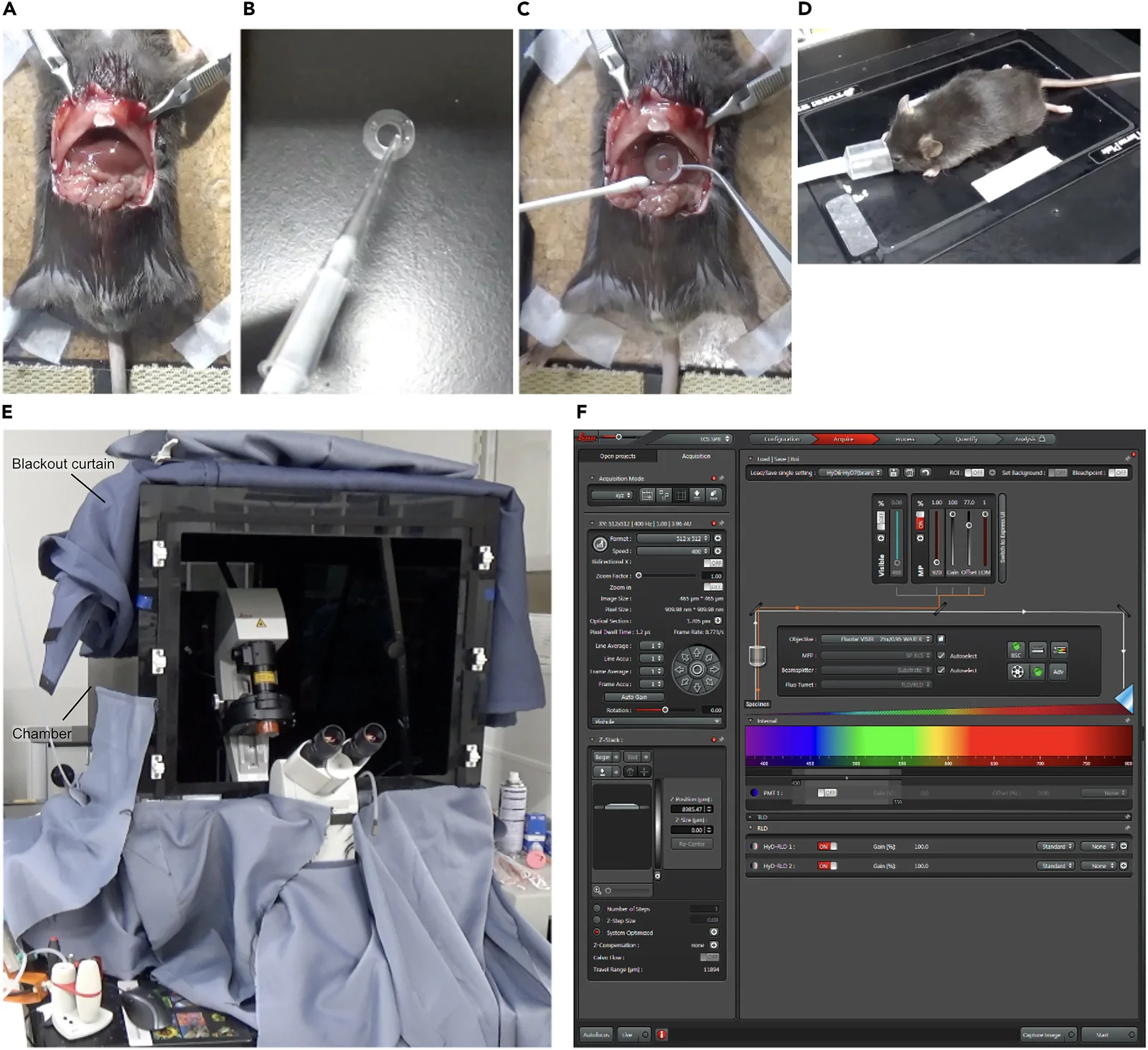
Preparation and setup for microimaging of the liver (A) The abdominal wall was opened to expose the liver. (B) Instant adhesive is applied to a polycarbonate washer with a pipette. (C) The washer is attached to the surface of the liver. (D) The mouse is placed in the prone position on the stage, with the washer positioned on a glass coverslip mounted on the stage heater. (E) The two-photon microscope setup. The chamber and blackout curtain are indicated. (F) Representative application window of Las X for two-photon imaging.
34. Apply a minimal amount of tissue-grade cyanoacrylate instant adhesive to one side of the polycarbonate washer using a P20 Pipetman equipped with a standard 200 μL pipette tip (Figure 3B).
35. Firmly attach the adhesive-coated side of the polycarbonate washer onto the targeted region of interest on the liver surface (Figure 3C).

### Imaging at cellular level using a two-photon microscope

**Timing: Variable (depending on duration of time-lapse imaging)**

This section describes the cellular-level imaging workflow using a two-photon microscope system. Compared with single-photon microscopy, two-photon excitation with near-infrared light penetrates deeper into scattering tissue such as the liver and confines fluorophore excitation to the focal plane, thereby reducing out-of-focus photobleaching and phototoxicity during time-lapse imaging. These properties enable stable monitoring of the FRET/GFP ratio at single-cell resolution in the intact organ.

Two-photon microscopy is not strictly required for ATP imaging; single-photon confocal systems, such as spinning-disk microscopes, can also be used for superficial tissue layers. In this case, the two emission channels should be acquired simultaneously to avoid misregistration caused by tissue motion.

36. Power on the microscope controller, host PC, Las X, and Mai Tai HP Ti laser system.
37. Configure the initial imaging parameters within the software:
  a. Excitation wavelength: 920 nm.
  b. Format: 512 × 512 pixels.
  c. Pixel size: 909.98 × 909.98 nm.
  d. Depth: 12-bit.
  e. Zoom: 1.0×.
  f. Pixel dwell time: 1.2 μs.
  g. Frame rate: 0.773/s (no frame or line averaging).

***Note:*** The 920 nm excitation wavelength is near the two-photon absorption maximum of EGFP, providing efficient excitation of the donor fluorophore.

***Note:*** These scanning parameters are suited to ATP changes occurring over minutes. In our experience, increasing the scanning speed to ∼30 frames/s (e.g., via resonant scanning) substantially reduces pixel dwell time and photon collection, resulting in noisy ratiometric images; thus, slower scanning rates (e.g., 0.773 frames/s) are preferable for stable quantification.

38. Confirm that the emitted fluorescence is split into two channels and detected with hybrid detectors (HyD): 525/50 nm for the EGFP channel and 585/40 nm for the FRET channel.
39. Select the appropriate objective lens depending on the desired field of view: 25× water-immersion objective lens (HC FLUOTAR L25×/0.95 W VISIR).
40. Securely attach a glass coverslip onto the stage heater unit.
41. Carefully apply a tiny droplet of cyanoacrylate instant adhesive to the exposed upper face of the polycarbonate washer (the side opposite to the liver tissue).
42. Maintaining continuous inhalation anesthesia via the nose cone, gently invert and position the mouse so that the washer attached to the liver rests directly on top of the coverslip, thereby bonding the washer firmly to the glass (Figure 3D).

***Note:*** The nose cone was custom-made from a 5-mL TERUMO syringe by cutting the syringe barrel approximately 3 cm from the needle-attachment end.

43. Adjust the XY position of the stage to center the mounted liver washer beneath the objective lens.
44. Seal the environmental chamber surrounding the two-photon microscope and drape it completely with a blackout curtain to exclude ambient light (Figure 3E).
45. Turn on the integrated epifluorescence illumination of the TCS SP8 MP using the RFP filter set to locate structural landmarks.
46. Select the exact region of interest and precisely adjust the z-position depth until active blood flow inside the hepatic sinusoid becomes clearly visible.

***Note:*** Imaging is typically performed at a depth of approximately 40 μm below the liver surface.

47. Turn off the epifluorescence illumination before starting two-photon image acquisition.
48. Optimize the detector gain to 100% and set the multiphoton laser power (typically 8% of the maximum output as set in the software) to obtain sufficient signal without causing tissue damage (Figure 3F).
49. Program the time-lapse acquisition parameters if tracking dynamic changes.

***Note:*** A 60-min imaging session at an acquisition interval of one frame per minute is a representative timeframe for tracking dynamic ATP changes (e.g., hepatic ATP monitoring during acute myocardial infarction^1^ ).

50. Initialize the time-lapse imaging session.

### Image analysis

**Timing**: **5 min/image**

This section describes the image analysis workflow. Fluorescence intensities are measured in ImageJ and FRET/GFP ratio values are calculated numerically in Excel, whereas ratio images for visualization are generated in MetaMorph.

51. Open the raw fluorescence image file in ImageJ.
52. Correct any potential XY spatial drift across time-lapse frames by executing the StackReg plugin (optional).
  a. Open all sequential time-lapse image frames in ImageJ by dragging and dropping the files into the window.
  b. Convert the individual frames into a single image stack via Image > Stacks > Images to Stack.
  c. Launch the plugin via Plugins > Registration > StackReg (or Plugins > StackReg depending on the installation path).
  d. In the transformation dropdown menu, select the appropriate alignment mode: Translation, Rigid Body, Scaled Rotation, Affine.
  e. Click OK to execute the alignment across all slices.
  f. Visually inspect the registered image stack and save the aligned stack via File > Save As > Tiff.
53. For macroscopic images, split the dual-channel image into separate EGFP and FRET channel images.
  ***Note:*** This step is not required for two-photon images, in which the two channels are acquired separately by independent detectors and are already stored as separate channels.
  a. Note the image width in pixels (Image > Properties…); the EGFP and FRET channels occupy the left and right halves of the image, respectively.
  b. Draw a rectangular selection over the left half of the image using the Rectangle tool (width, half the image width; height, the full image height; X coordinate, 0; Y coordinate, 0).
  c. Duplicate the selected region with Image > Duplicate… (check “Duplicate stack” for time-lapse series) and name it “EGFP”.
  d. Repeat steps b and c for the right half of the image, setting the X coordinate to half the image width, and name the duplicated image “FRET”.
54. Manually outline and define the desired regions of interest over the target organs or cellular structures.
  a. Using the selection tools, outline the region to be analyzed in the EGFP channel image.
  b. Add it to the ROI Manager (Analyze > Tools > ROI Manager, then click “Add”).
55. Quantify the mean fluorescent intensities for both the EGFP channel and the Kusabira Orange channel.
  a. Go to Analyze > Set Measurements, and check “Mean gray value.”
  b. Select the ROI in the ROI Manager, click “Measure”, and record the mean gray value.
  c. Activate the FRET channel image, select the same ROI in the ROI Manager, and click “Measure” to obtain the mean gray value at the identical coordinates.

***Note:*** The same ROI must correspond to the identical anatomical region in both channels.

56. Export the raw numbers and compute the final FRET/GFP intensity ratios using Microsoft Excel.

***Note:*** The ratio is calculated by dividing the mean intensity of the FRET channel by the mean intensity of the EGFP channel for the same ROI (FRET/GFP ratio = mean intensity of the FRET channel ÷ mean intensity of the EGFP channel).

***Note:*** Background subtraction is not performed in this workflow. In AVID mice, GO-ATeam2 fluorescence is at least 7-fold higher than tissue autofluorescence, a criterion used in establishing the AVID line. Under this condition, the contribution of background signal to both emission channels is negligible: in our primary study, ATP levels estimated from FRET/GFP ratios without background subtraction agreed with parallel luciferase-based ATP measurements, whereas signals below this threshold produced large deviations (Figure S16 of Ohnishi et al^1^).

57. Open both the raw FRET channel image and the GFP channel image within MetaMorph offline software to construct pseudocolored ratio images.
58. Launch the “Ratio Images” module in MetaMorph and designate the FRET image as the Numerator and the GFP image as the Denominator.
59. Check the “All planes” option to process the entire time-lapse stack. (optional).
60. Set the display configuration to “IMD display” (8 ratio-32 intensities mode).
61. Define the minimum and maximum ratio thresholds depending on the acquisition modality: set the Minimum ratio to 0.8 and the Maximum ratio to 3.0 for our macroscopic images, or set the Minimum ratio to 0.4 and the Maximum ratio to 2.0 for our two-photon microscopic images.
62. Render the final structural visualization by selecting “As Displayed” with the target ratio image designated as the source, then save the resulting pseudocolored FRET ratio images.

## Expected outcomes

Using the macroscopic imaging configuration of this protocol with the 0.63× objective lens at a zoom factor of 0.73×, intra-abdominal and intrathoracic organs can be comprehensively monitored within a single field of view in adult mice (Figure 4A). Surgical trauma to the organ induces an artificial decline in ATP levels (Figures 4A and 4B). The FRET/GFP ratio is characteristically higher in the heart and skeletal muscle, while displaying lower levels in the small intestine.

**Figure 4:**
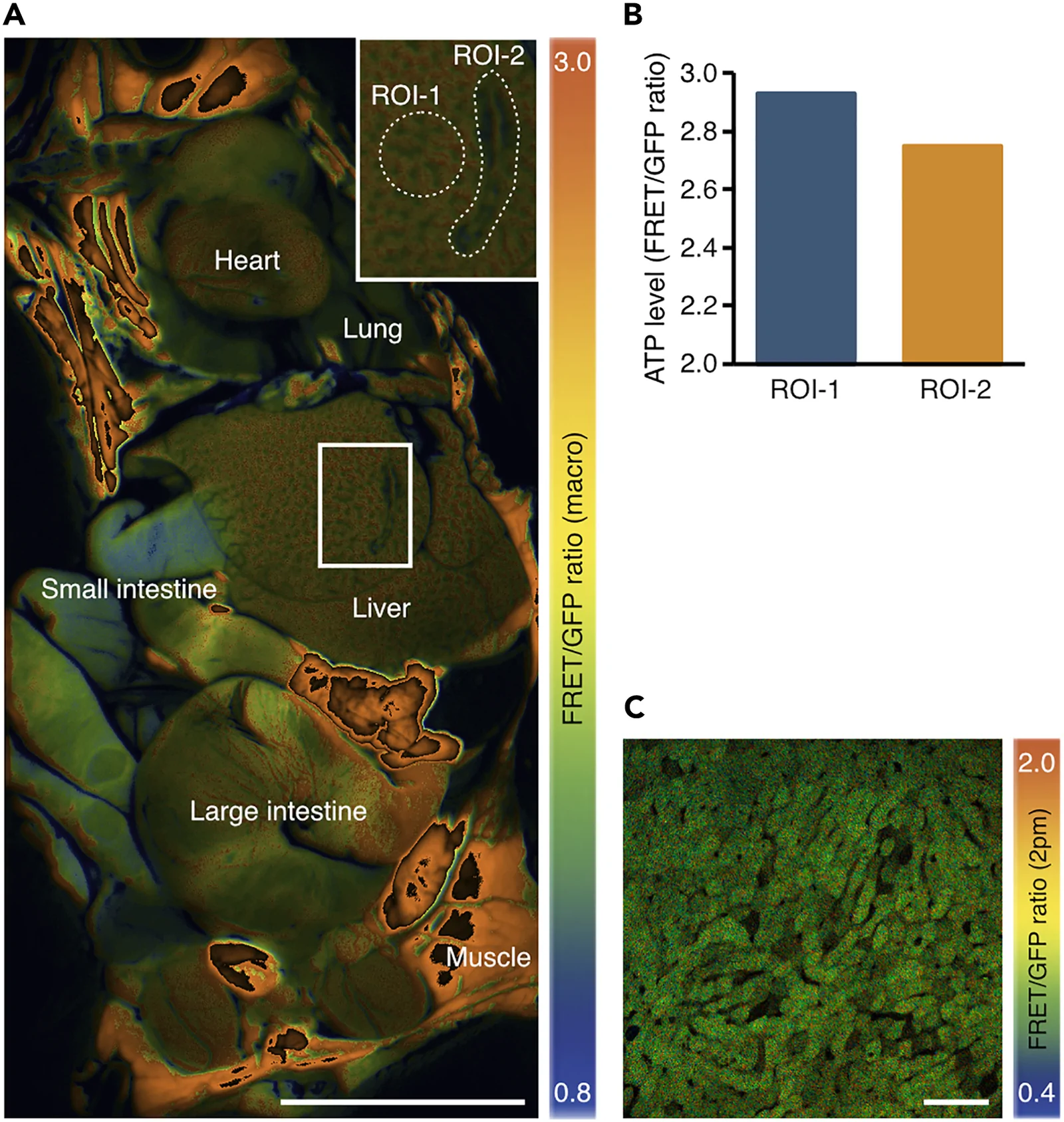
Representative images obtained with a macroscope and a two-photon microscope (A) Intravital ATP imaging of intrathoracic and abdominal organs using a macroscope (macro) in an adult mouse. The rectangle in the liver represents the enlarged image shown in the inset. The dotted line outlines the intact area (ROI-1) and area of surgical trauma (ROI-2). The color bar indicates ATP levels represented by the FRET/GFP ratio, ranging from 0.8 (blue, lower ATP levels) to 3.0 (red, higher ATP levels). Scale bar, 1 cm. (B) Quantification of the FRET/GFP ratios of ROI-1 and ROI-2 in the panel A. The FRET/GFP ratio is lower in the area of surgical trauma (ROI-2) than in the intact area (ROI-1). (C) Intravital ATP imaging of the liver using a two-photon microscope (2pm) in an adult mouse. The color bar indicates ATP levels represented by the FRET/GFP ratio, ranging from 0.4 (blue, lower ATP levels) to 2.0 (red, higher ATP levels). Scale bar, 100 μm.

Two-photon imaging executed via this protocol successfully tracks the FRET/GFP ratio at single-cell resolution within the liver of adult mice (Figure 4C). When processed with a minimum ratio threshold of 0.4 and a maximum ratio threshold of 2.0, hepatocytes demonstrate a structurally uniform and clear FRET/GFP ratio distribution across all parenchymal regions under physiological baseline conditions. Although the liver is presented here as a representative organ, the two-photon imaging approach is not restricted to hepatic tissue; for example, cellular-level ATP imaging of the small intestine has been demonstrated in our primary study.^1^ In principle, any organ surface that can be surgically exposed and stabilized in the same manner is suitable for this workflow.

Furthermore, this two-photon imaging protocol is highly capable of tracking dynamic metabolic shifts in real-time. For instance, in a mouse model of acute myocardial infarction, continuous imaging of the liver clearly captures a progressive decline in the FRET/GFP ratio, indicating ATP depletion, which begins approximately 10 min after ligation and is spatially restricted to the pericentral region surrounding the central vein (Video S6 of Ohnishi et al.^1^).

## Limitations

Genetically encoded fluorescent biosensors, including GO-ATeam2, can be inherently influenced by environmental factors such as oxygen concentration, pH, and temperature. In our previous study,^1^ we experimentally validated that two major potential confounders—oxygen tension and pH—have a minimal impact on the FRET/GFP ratio under our presented imaging conditions. For instance, in skeletal muscle, a 10 s stimulation at 100 Hz did not significantly alter local tissue oxygenation or pH, indicating that the tracked ATP depletion represents genuine metabolic shifts rather than sensor artifacts (Figures S6 and S7 of Ohnishi et al^1^; ). Similarly, in the liver, the FRET/GFP ratio remained stable despite hypoxia (under 10% O_2_ ventilation) and mild acidosis (a decrease of ∼0.4 pH units induced via lactate injection), demonstrating the ratiometric robustness of the GO-ATeam2 sensor against mild physiological environment alterations (for the experimental data and representative images, see Figures S13 and S14 of Ohnishi et al^1^; ). However, users must be aware that more severe or prolonged hypoxic and acidotic conditions may still perturb the biosensor behavior. In addition, peripheral oxygen saturation was not measured in this protocol. Because hypoxemia resulting from inadequate ventilation directly affects intracellular ATP levels, we recommend monitoring peripheral oxygen saturation when feasible, particularly during prolonged imaging sessions.

Other physicochemical factors, such as reactive oxygen species (ROS) or rapid ionic fluctuations, have not been systematically evaluated in vivo and could potentially affect the fluorescence readouts. Therefore, the interpretation of FRET signals under extreme pathophysiological states should be approached with caution.

Furthermore, temperature management is critical when executing this protocol; the sensitivity of GO-ATeam2 can be affected under hypothermic conditions. Lower ambient or core body temperatures, such as during cold exposure or due to insufficient stage heating, could alter the conformational dynamics of the ε subunit and modify the fluorescence properties of the FRET pair, thereby confounding quantitative readouts. Thus, maintaining the mouse core body temperature at a physiological level on a 37°C heat pad is strictly required, and any ATP imaging under hypothermic contexts must be interpreted with extreme caution. Of note, core body temperature was not directly measured in this protocol; the heat pad setting alone does not ensure maintenance of physiological core temperature. Therefore, we recommend monitoring rectal temperature when feasible, especially for prolonged imaging sessions or under conditions in which thermoregulation may be compromised.

Additionally, although GO-ATeam2 is robustly expressed across all tissues in the AVID mouse, the absolute expression levels vary considerably by organ, showing an up to ∼5-fold variation in Kusabira Orange direct fluorescence intensity and an up to ∼20-fold difference in protein expression levels. While these expression variations do not substantially skew individual ATP measurements due to the self-normalizing ratiometric design, users should be cautious of potential biases when directly comparing tissues with markedly different architectural features or baseline biosensor expression profiles.

Finally, while the *in vivo* physiological and pathological models presented in our primary study predominantly captured decreases in ATP levels, the dynamic capacity of this system to sensitively detect acute ATP elevations has been validated. Specifically, we confirmed that this platform successfully monitors and quantifies real-time ATP increases in preimplantation embryos (two-cell stage embryos) upon the administration of a prodrug, proAX, in the culture medium, as detailed in Supplementary Figure 5 of our primary manuscript.^1^ These data allowed us to generate a Hill equation-based curve and estimate the *Kd* value of the ATP sensor within the cellular environment. Therefore, users can reliably apply this imaging protocol to investigate not only ATP depletion but also acute energy supplementation and metabolic surges across various biological contexts. Nevertheless, it should be emphasized that the standard workflow described in this protocol yields relative, ratiometric ATP readouts rather than absolute intracellular ATP concentrations. Converting FRET/GFP ratios into absolute ATP concentrations would require an in vivo calibration procedure, including the determination of the maximal and minimal ratios of the sensor in each tissue and imaging configuration, which is not covered by the present protocol and remains technically challenging in intact organs. Users should therefore interpret the FRET/GFP ratio as a relative index of intracellular ATP levels and restrict quantitative comparisons to those made within the same tissue and imaging conditions.

## Troubleshooting

### Problem 1

GO-ATeam fluorescence signal intensity is insufficient or uneven across different organs during macroscopic imaging due to tissue-specific baseline expression variability. Related to Steps 19 and 22.

### Potential solution

Separately optimize and adjust the excitation light power for each specific target organ, although this prevents simultaneous imaging of multiple organs in the same field. Prior to data collection, experimentally validate that the calculated FRET ratio remains stable and unperturbed by variations in the applied excitation light power.

### Problem 2

The mouse exhibits breathing artifacts or subtle body movements during the imaging session. Related to Steps 11 and 31.

### Potential solution

The physical immobilization of the animal may be inadequate. Ensure that the upper incisors are securely suspended and the tail is fixed tightly with high-adhesion tape to completely eliminate any displacement along the rostrocaudal axis. Furthermore, prolonged imaging can cause spontaneous shifting or drifting of intra-abdominal organs. To counteract this internal organ movement, apply a minimal amount of cyanoacrylate instant adhesive to the non-visible under-surfaces (backside) of the target organs to anchor them securely to surrounding stable structures away from the active imaging field.

### Problem 3

The fluorescence signal drops drastically or the fine cellular structures become obscure during two-photon liver imaging. Related to Step 42.

### Potential solution

An air gap or pocket may have formed in the light path between the glass coverslip sample window and the water-immersion objective lens. Gently add saline or water pre-warmed to 37°C from the side to completely eliminate the gap and bridge the optical path. Alternatively, excessive spreading of the cyanoacrylate instant adhesive onto the active window area of the polycarbonate washer can severely distort the tissue architecture and cloud visibility. If adhesive contamination occurs, carefully remove the washer and re-attach a fresh polycarbonate washer onto a clean site on the liver.

### Problem 4

The endotracheal intubation procedure fails to achieve proper mechanical ventilation, and the chest wall does not move in sync with the ventilator pump cycles. Related to Step 8.

### Potential solution

The cannula has likely been misrouted into the esophagus rather than entering the trachea. Promptly remove the cannula, perform the endotracheal intubation sequence again from the beginning, and visually confirm perfect synchronization between the thoracic wall movement and the ventilation equipment.

### Problem 5

Upon rendering the pseudocolored ratio image, a localized, abnormal drop in the FRET/GFP ratio is observed within an otherwise healthy organ structure. Related to Steps 13, 14, 15, and 32.

### Potential solution

This artifact is frequently caused by mechanical trauma during laparotomy or thoracotomy, such as pinching or pulling the tissue roughly with surgical forceps. Never grasp or compress live organs with metal forceps. Use only soft, lint-free cotton swabs to gently reposition organs if necessary, and strictly avoid touching or manipulating the active upper surface intended for optical observation.

### Problem 6

During extended, long-term imaging sessions, the global ATP levels across the entire animal begin to degrade and decline. Related to Steps 28 and 50.

### Potential solution

Ensure that the depth of anesthesia is properly maintained to avoid excessive physiological depression. In our preliminary setup, maintaining inhalation anesthesia at 1.5%–2.0% isoflurane supported a physiological heart rate (typically ≥480 bpm, confirmed by preliminary electrocardiogram recordings) without accidental awakening. If monitoring equipment (e.g., electrocardiogram) is available, users may monitor the heart rate to ensure that it remains within this physiological range; otherwise, carefully titrating the isoflurane concentration to the minimal required level (1.5%–2.0%) is recommended to avoid bradycardia-induced ATP depletion.

Tissue surface desiccation can cause stress and drop the ratio; routinely spray a fine mist of sterile physiological saline onto the exposed organs using an atomizer, or administer a slow continuous intravenous infusion of saline via the jugular vein. When not actively scanning, cover the exposed surgical cavities with plastic wrap to preserve local moisture. Additionally, check the temperature stability of the warming stage; poor temperature control loops or direct draft from laboratory air conditioning vents can quickly cool and weaken the animal. Shield the surgery area from ambient wind and maintain ambient warmth.

## Resource availability

### Lead contact

Requests for further information, resources, and reagents should be directed to and will be fulfilled by the lead contact, Masamichi Yamamoto (myamamoto@ncvc.go.jp).

### Technical contact

Technical questions on executing this protocol should be directed to and will be answered by the technical contact, Masamichi Yamamoto (myamamoto@ncvc.go.jp).

### Materials availability

AVID mice have been deposited in The Jackson Laboratory under JAX stock no. 041022. Distribution of the mouse strain requires a standard material transfer agreement (MTA).

### Data and code availability

The published article includes all datasets generated or analyzed during the primary study. No custom computer codes were generated for this protocol.

## Acknowledgments

This work was supported by 10.13039/501100001691JSPS 10.13039/501100001691KAKENHI (grant nos. 24K20531 and 26KJ0437 to R.O.), 10.13039/501100001695JST 10.13039/501100009023PRESTO (grant no. JPMJPR14MF to M.Y.), JSPS KAKENHI (grant nos. JP24116703, 24657144, 26670100, 19K22513, 19H03561, and JPMJPF2018 to M.Y.), 10.13039/100009619AMED (grant nos. JP17gm5010002, 23ama121050j0002, 20ek0610017h0003, and 21gm1210011h0002 to M.Y.), the 10.13039/100007428Naito Foundation, the Mitsui Life Social Welfare Foundation, the 10.13039/100007449Takeda Science Foundation, the 10.13039/501100004632Mother and Child Health Foundation, the 10.13039/100008732Uehara Memorial Foundation, the 10.13039/501100008643Japan Brain Foundation, the Nakajima Foundation, the 10.13039/501100008642Foundation for Dietary Scientific Research, the 10.13039/501100012036Lotte Foundation, a 10.13039/501100005865Mochida Memorial Foundation for Medical and Pharmaceutical Research grant, the 10.13039/501100003348Fukuda Foundation for Medical Technology, the 10.13039/100016289Taiju Life Social Welfare Foundation, the Narishige Neuroscience Research Foundation, the 10.13039/501100008673Yasuda Medical Foundation, the 10.13039/501100007263Astellas Foundation for Research on Metabolic Disorders, the 10.13039/501100008667SENSHIN Medical Research Foundation, the 10.13039/501100004088Kowa Life Science Foundation, the Miyata Cardiac Research Promotion Foundation, the Shimadzu Science Foundation, the 10.13039/501100008884Nakatani Foundation for Advancement of Measuring Technologies in Biomedical Engineering, the 10.13039/501100012018Research Foundation for Opto-Science and Technology, 10.13039/100007716Banyu Life Science Foundation International, the 10.13039/501100005936Casio Science Promotion Foundation, the 10.13039/100007434Suzuken Memorial Foundation, the 10.13039/501100004051Kato Memorial Bioscience Foundation to M.Y., and the Intramural Research Fund (21-2-7 and 22-B-3 to M.Y.) for Cardiovascular Diseases of the 10.13039/100016924National Cerebral and Cardiovascular Center.

## Author contributions

R.O. and M.Y. wrote, reviewed, and edited the manuscript.

## Declaration of interests

M.Y. holds patents related to the GO-ATeam mice that have been granted in Japan and the United States.
